# Multi-Locus Assortment (MLA) for Transgene Dispersal and Elimination in Mosquito Populations

**DOI:** 10.1371/journal.pone.0005833

**Published:** 2009-06-08

**Authors:** Jason L. Rasgon

**Affiliations:** 1 The W. Harry Feinstone Department of Molecular Microbiology and Immunology, Bloomberg School of Public Health, Johns Hopkins University, Baltimore, Maryland, United States of America; 2 The Johns Hopkins Malaria Research Institute, Baltimore, Maryland, United States of America; BMSI-A*STAR, Singapore

## Abstract

**Background:**

Replacement of wild-type mosquito populations with genetically modified versions is being explored as a potential strategy to control vector-borne diseases. Due to lower expected relative fitness of transgenic individuals, transgenes must be driven into populations for these scenarios to be successful. Several gene drive mechanisms exist in a theoretical sense but none are currently workable in mosquitoes. Even if strategies were workable, it would be very difficult to recall released transgenes in the event of unforeseen consequences. What is needed is a way to test transgenes in the field for feasibility, efficacy and safety prior to releasing an active drive mechanism.

**Methodology/Principal Findings:**

We outline a method, termed Multi-locus assortment (MLA), to spread transgenes into vector populations by the release of genetically-modified mosquitoes carrying multiple stable transgene inserts. Simulations indicate that [1] insects do not have to carry transgenes at more than 4 loci, [2] transgenes can be maintained at high levels by sequential small releases, the frequency of which depends on the construct fitness cost, and [3] in the case of unforeseen negative non-target effects, transgenes can be eliminated from the population by halting transgenic releases and/or mass releases of wild-type insects. We also discuss potential methods to create MLA mosquito strains in the laboratory.

**Conclusions/Significance:**

While not as efficient as active drive mechanisms, MLA has other advantages: [1] MLA strains can be constructed for some mosquito species with currently-available technology, [2] MLA will allow the ecological components of transgenic mosquito releases to be tested before actual gene drive mechanisms are ready to be deployed, [3] since MLA is not self-propagating, the risk of an accidental premature release into nature is minimized, and [4] in the case that active gene drive mechanisms prove impossible to develop, the MLA approach can be used as a back-up transgene dispersal mechanism for disease control efforts in some systems.

## Introduction

Vector-borne diseases such as malaria and dengue are a significant re-emerging public health threat [1]–[4]. There are no currently available vaccines to control malaria or dengue, and control is limited to anti-parasitic drugs (in the case of malaria) and control of the relevant mosquito vectors [5]. Since traditional methods and their implementations have failed to keep vector-borne diseases in check, there has been much interest in applying new molecular biological advances for vector-borne disease control [6]–[14]. One proposed method is to replace competent mosquito populations with those refractory to pathogen transmission. Transgenic manipulation of mosquitoes to alter their ability to transmit pathogens them is now a reality [10], [12] and the time has come to think critically about the best method to deploy this technology in the field.

To be successful, most population replacement strategies for vector-borne disease control require introduced transgenes to spread to high frequency in populations [14]–[15]. This is not expected to occur spontaneously. Several methods for gene drive under consideration include *Wolbachia* symbionts [11], [16]–[17], fitness manipulation [18], transposable elements (TE's) [7], [13], MEDEA [9] and homing endonucleases (HEGs) [19]. While these strategies have been examined in theory, and proof-of-principle demonstrated for some systems in *Drosophila*, none are currently workable in mosquitoes.

What is needed is a way to test potential transgene constructs in the field for feasibility, efficacy and safety prior to releasing an active drive mechanism. As constructs are being developed now, a method that relies on currently available technology would be desirable. This method should be able to adequately spread introduced traits into populations at logistically low release levels (feasibility) spread the transgene to a high enough frequency to interrupt pathogen transmission cycles (efficacy) and demonstrate the absence of unforeseen non-target effects (safety). If unforeseen negative consequences arise, the introduced transgene constructs should be recallable or eliminated from the population.

We outline herein a method (Multi-locus assortment; MLA) to spread transgenes into vector populations by gene “diffusion” rather than gene drive. This method requires the creation of genetically-modified mosquito (GMM) strains carrying “stacked” or multiple stable transgene inserts. By a combination of transgenesis technology and classical crossing methods, mosquito strains that are homozygous for 4 transgene loci (8 total inserts) are developed. When released into cage or wild populations, the transgene inserts assort into the population by recombination. Over time, the average number of transgenes that any one individual carries decreases, but the frequency of individuals carrying at least one transgene dramatically increases. The transgene can be maintained at high frequency in the population by additional, relatively infrequent releases (every 5–10 generations). Using appropriate outcrossing procedures, it should be possible to maintain genetic diversity in the release strain. As transgene inserts will not be mobile, fitness costs per insert should be substantially less than those caused by mobile transposable elements [20]. By careful selection of transgenic lines, fitness effects can be minimized even further.

While not as theoretically efficient as active gene drive mechanisms, the MLA approach has several advantages that make it a potentially attractive strategy for consideration. All technical requirements for the generation and application of MLA mosquito strains currently exist, and there are no empirical or theoretical barriers to the development and application of this technology. MLA strains would allow the ecological components of transgenic mosquito releases to be tested before actual gene drive mechanisms are ready to be deployed. Since MLA is not self-propagating, the risk of an accidental premature release into nature is minimized. Finally, in the case that active gene drive mechanisms prove impossible to develop, the MLA approach can be used as a back-up transgene dispersal mechanism for disease control efforts.

Theory has been developed to follow the spread of some types of multi-locus traits, but these efforts focused on the spread of conditional lethal or female-killing genes, and were based on lepidopteran genetics (up to 20 chromosomes) [21]–[22]. In this paper, we explore this strategy using simple population genetics models based on mosquito genetics. We also discuss several strategies to construct MLA mosquito strains in the laboratory.

## Results

**Figure 1:**
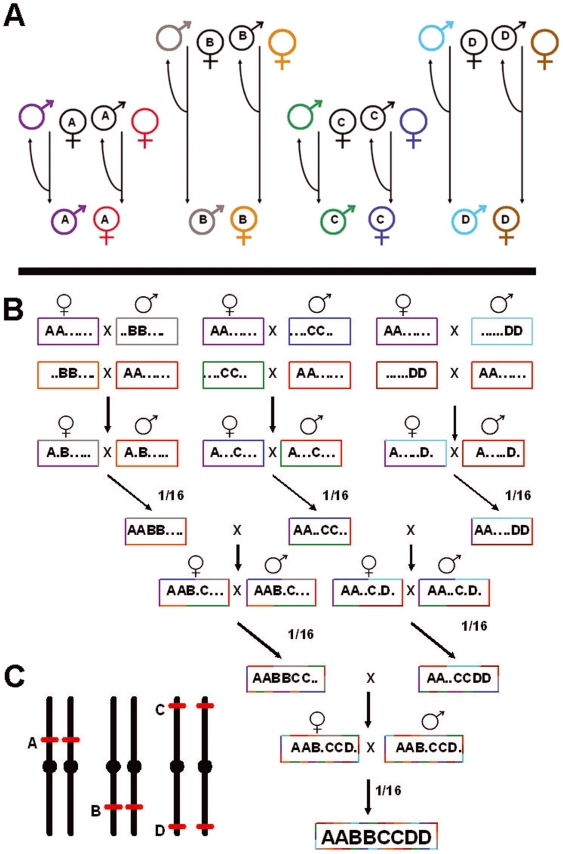
Crossing scheme to create an MLA mosquito strain. A: Each transgenic line (A–D) is outcrossed to one of two wild-type backgrounds (denoted by colors). The final 8 outcrossed transgenic lines are made homozygous. B: The 8 outcrossed transgenic lines are specifically crossed and offspring of the correct genotype selected each generation to construct the MLA strain. Correct genotyping of offspring can potentially be automated in a high-throughput manner (see text and Figure 8). C: Hypothetical locations of each transgenic locus so that all 4 loci assort independently.

The modeling framework can be used to explore diverse MLA strategies for transgene spread and elimination. The model simulations also present hypotheses that can be empirically verified or refuted by cage experiments. As examples, we outline 3 different situations to explore the utility of MLA for driving transgenes into mosquito populations, and 2 different strategies to eliminate the transgene from the population in the advent of unforeseen negative consequences. For simplicity, the simulations below assume that MLA mosquito strains are constructed that carry transgenes at 4 unlinked loci. This could be accomplished by placing 2 transgenes on separate linkage groups and having the third and fourth transgene on the same linkage group but separated by a significant distance (such as opposite chromosomal arms) (Figure 1).

### Situation 1. Gene drive; Single mass release

**Figure 2:**
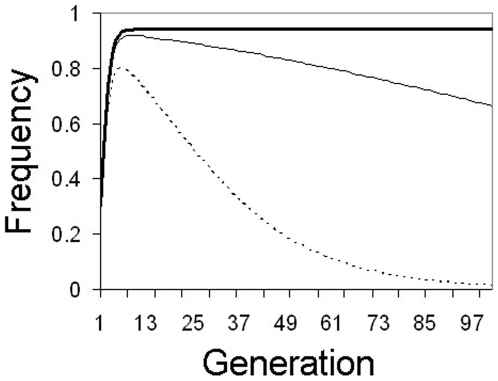
Single mass release. Changes in transgene frequency after a single 30% release of mosquitoes homozygous for a transgene at 4 unlinked loci (8 copies/genome). Thick solid line: No fitness cost (*F* = 1.0), thin solid line: 1% fitness cost/insert (*F* = 0.99), thin dotted line: 5% fitness cost/insert (*F* = 0.95).

The goal of a gene drive system is to maximize the efficiency of transgene spread while minimizing the number of insects that must be released. Inundative release of mosquitoes homozygous for a stable insert at a single locus is impractical due to the very high numbers of insects that must be released. Placing multiple independently-assorting inserts within the released mosquitoes' genomes can make this strategy more logistically feasible because a greater than 50% proportion of offspring inherit at least one transgene for multiple subsequent generations. If mosquitoes are released that are homozygous at 4 loci, a single mass release of 30% can result in over 94% of the mosquitoes in the population possessing at least one copy within 8 generations post-release if the insert does not induce fitness costs. If transgene inserts are costly, the frequency of individuals carrying ≥1 copy will increase initially then decrease over time as selection eliminates the transgene from the population. The rate of decrease depends on the strength of induced fitness costs. If costs are small (≤1%/insert), high frequencies (≥80%) can still be maintained even 60 generations after the release. Higher fitness costs will result in faster elimination (Figure 2).

### Situation 2a. Gene drive; Continuous release – 1% per generation

If transgenic manipulation does not pose significant fitness costs to mosquitoes, MLA may allow the deployment of transgenic mosquitoes with only a few or even a single moderately large release (see above). However, it is reasonable to assume that there will be some fitness load experienced, either by the disruption of critical genes or regulatory regions by the transgene, or by expression of the effector molecule. In the case of fitness costs, single mass releases may not be sufficient to introduce and/or maintain the transgene at a high enough frequency to interrupt pathogen transmission cycles. Thus, one may choose to release transgenic mosquitoes every generation. MLA will improve the efficacy of this type of continuous release strategy. Even if transgenes are neutral, a continuous small release strategy may be more logistically feasible than a single mass release depending on the biology of the specific system.

**Figure 3:**
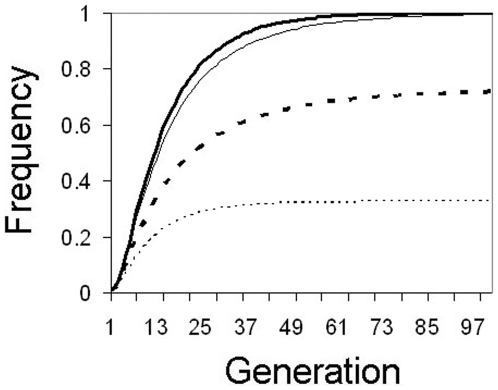
Continuous minimal release. Changes in transgene frequency with 1% continuous release rate per generation of mosquitoes homozygous for a transgene at 4 unlinked loci (8 copies/genome). Thick solid line: no fitness cost (*F* = 1.0), thin solid line: 1% fitness cost/insert (*F* = 0.99), thick dotted line: 5% fitness cost/insert (*F* = 0.95), thin dotted line: 10% fitness cost/insert (*F* = 0.9).

If transgene inserts are neutral or cause low (≤1% per insert) fitness costs, a 1% per generation continuous release rate of 4-locus homozygotes is sufficient for rapid transgene spread to high frequency (>90% in less than 40 generations), approaching fixation in 60–70 generations. If transgenes cause larger fitness costs (≥5% per insert), transgenes can be maintained in the population, but equilibrium frequencies will be appreciably less than fixation (Figure 3).

### Situation 2b. Gene drive; Continuous release – 5% generation

**Figure 4:**
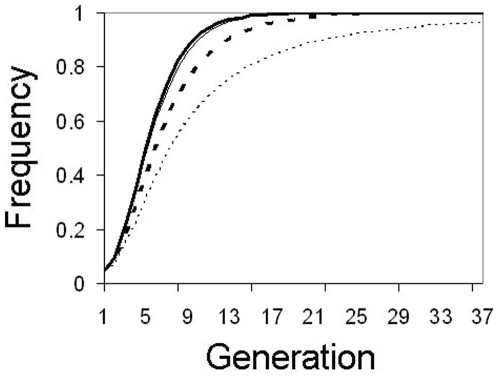
Continuous moderate release. Changes in transgene frequency with a 5% continuous release rate per generation of mosquitoes homozygous for a transgene at 4 unlinked loci (8 copies/genome). Thick solid line: no fitness cost (*F* = 1.0), thin solid line: 1% fitness cost/insert (*F* = 0.99), thick dotted line: 5% fitness cost/insert (*F* = 0.95), thin dotted line: 10% fitness cost/insert (*F* = 0.9).

Slightly higher release rates can significantly increase the efficiency of a continuous release MLA strategy. If 4-locus homozygotes are introduced at a higher continuous release rate of 5% per generation, the frequency of individuals carrying ≥1 copy can rapidly increase to very high frequency, reaching ≥96% in 40 generations even in the case of large induced fitness costs of up to 10% per insert. More moderate fitness costs (≤5% per insert) can result in transgene frequencies approaching fixation in approximately 20 generations (Figure 4).

### Situation 3. Gene drive; Discontinuous release – 5% every 10 generations

**Figure 5:**
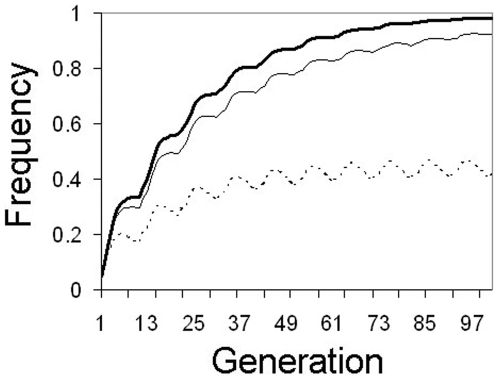
Discontinuous release. Changes in transgene frequency with a 5% release every 10 generations of mosquitoes homozygous for a transgene at 4 unlinked loci (8 copies/genome). Thick solid line: no fitness cost (*F* = 1.0), thin solid line: 1% fitness cost/insert (*F* = 0.99), thin dotted line: 5% fitness cost/insert (*F* = 0.95).

We have shown that, in theory, MLA makes it possible to spread transgenes into populations in spite of even moderate to heavy fitness costs (5–10% per insert) using continuous low-level releases. However, the efficiency advantage of MLA may make this unnecessary. Because one gets multiple generations of gene spread from a single release (due to assortment of the multiple loci) it may be possible to reduce the number of releases that need to be performed. For example, by releasing 4-locus homozygotes at 5% every 10 generations, transgenes that cause low fitness costs (≤1% per insert) can spread into the population and reach over 80% within 40 generations. Transgenes inducing higher fitness costs can be maintained in the population, but will equilibrate at frequencies appreciably lower than fixation. In this case, releases will have to be at a higher release rate and/or performed more often. However, it is clear that depending on the fitness characteristics of the transgene, MLA can make discontinuous release a potentially feasible method to drive genes into populations (Figure 5).

### Situation 4. Passive transgene elimination; Stop releasing transgenic mosquitoes

**Figure 6:**
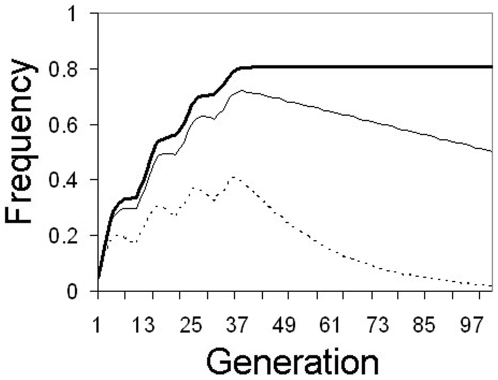
Passive transgene elimination. Changes in transgene frequency with a 5% discontinuous release every 10 generations of mosquitoes homozygous for a transgene at 4 unlinked loci (8 copies/genome). Releases are halted after the fourth release. If transgenes are costly, selection eliminates the construct from the population. Thick solid line: no fitness cost (*F* = 1.0), thin solid line: 1% fitness cost/insert (*F* = 0.99), thin dotted line: 5% fitness cost/insert (*F* = 0.95).

One major problem with many of the active drive mechanisms currently under consideration is that if the drive system works as required it will be extremely difficult to recall the construct after release in the case of unforeseen negative consequences (although some traits, such as HEGs, can be theoretically recalled by releasing insects carrying resistance alleles; 19). By design, MLA is self-limiting in nature. Halting releases when negative outcomes are detected is a passive strategy that does not require the outlay of further effort or resources. If transgene inserts are neutral, halting releases in the middle of the release campaign will result in the transgene maintaining an equilibrium frequency in the population. However, if inserts induce any fitness cost, selection will eliminate the transgene from the population over time. The speed of elimination is, intuitively, directly proportional to the fitness cost (Figure 6).

### Situation 5. Active transgene elimination; Release wild-type mosquitoes

Some researchers have argued that transgenic manipulation may not impose a fitness cost on mosquitoes, and that actual transgenic strains destined for release will be screened to minimize or eliminate negative fitness effects [12]. If this is the case, then strategies designed to spread genes into populations will work with maximum efficiency. However, it will also be much more difficult to eliminate the transgene from the population if it becomes necessary to do so. While passive transgene elimination may not be possible if constructs are neutral, MLA also offers an active transgene elimination option. In this case, release of transgenic mosquitoes is halted and wild-type individuals released instead, essentially diluting out the transgene.

**Figure 7:**
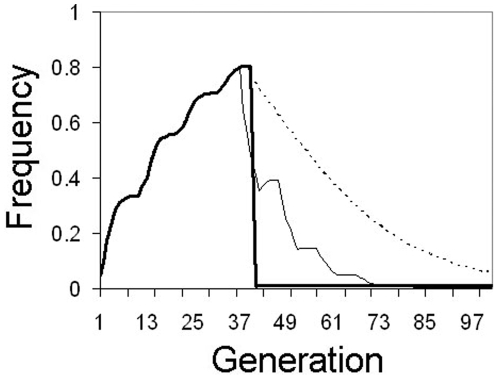
Active elimination of a neutral transgene (*F* = 1.0). 4-locus homozygous mosquitoes are initially released at a 5% frequency every 10 generations. At generation 40, transgenic releases are halted and the transgene eliminated by release of wild-type mosquitoes. Thick solid line: mass 99∶1 wild-type release, Thin solid line: 5 “burst releases” of wild-type at a rate of 20% for 5 generations separated by 5 generations, thin dotted line: continuous 5% wild-type release every generation.

There are several different methods that can be deployed to do this, and the choice of method will depend on the biology of the particular system. After halting release of transgenic mosquitoes, a single large 99∶1 mass release of wild-types is sufficient to drive transgene frequency from ∼80% to ∼1% (Figure 7). In many mosquito systems it may not be logistically possible to conduct a mass release of this magnitude, although some sterile insect release programs are able to do so due to the construction of industrial-scale rearing factories [23].

At the other extreme, sequential moderately low (5%) releases every generation is sufficient to drive transgene frequency to very low levels. A disadvantage of a low-level continuous release strategy is that it is slow, taking over 75 generations to drive transgene frequency from ∼80% to <1% (Figure 7). However, for mosquitoes with large population sizes, it may be the only feasible method.

In between these two extremes, there is another method that may be a good compromise between efficacy and feasibility. Using a “burst” release strategy, medium-sized releases are conducted for a number of generations then halted for a number of generations. This is repeated until transgene frequency has reached an acceptably low level. For example, by releasing wild-type mosquitoes at a rate of 20% per generation for 5 continuous generations, then waiting 5 generations before repeating, one can drive transgene frequency to <1% in 5 release regimes (Figure 7). Larger releases can be used to lower the number of sequential releases that must be performed, or smaller releases can be made when coupled with more release repetitions. The optimal protocol will depend on the biology of the species of interest.

### Potential strategies for constructing MLA mosquito strains

Maintaining genetic diversity during the creation of an MLA mosquito strain homozygous at 4 loci will be very challenging, but not impossible. We believe that this can in principle be accomplished by the crossing scheme outlined in Figure 1. Starting with 4 distinct transgenic strains (denoted by letters A–D), each carrying a transgene insert at a different genomic location, and 8 genetically distinct wild-type strains (denoted by color), transgenes are introgressed and 8 genetically distinct transgenic lines created and made homozygous for their respective transgene. These 8 lines are then intercrossed as shown, with offspring of the appropriate genotypes selected at each crossing. Within six rounds of crossing, a genetically diverse strain carrying all 8 inserts can be theoretically created.

In *Aedes aegypti*, strains have been produced that are homozygous at two loci (four inserts total) [24]. The crossing scheme to generate this strain was complicated by the background genetics of the insertion sites (homozygotes at one locus had a severe fitness cost in some lines) (Balakathiresan, pers. comm.) and difficulties in visually identifying heterozygotes from homozygotes, necessitating the generation and use of a PCR assay. The effort to create mosquito strains carrying more inserts increases with every locus added. It is clear that for MLA strains to be created, novel methods must be used to make the effort much less prohibitive.

Multiple *Aedes aegypti* strains containing phi C31 “docking sites” have been created (and additional strains can be generated in the future), allowing for the creation of multiple transgenic mosquito strains that carry the transgene at defined sites in the genome [25]. These strains can be screened beforehand to eliminate those with significant site-specific fitness effects. In addition, many different fluorescent marker proteins exist (green, red, yellow, and blue). In the crossing scheme outlined in Figure 1, it is necessary to distinguish individuals who are heterozygous vs. homozygous for each transgene to construct the MLA strain. In some transgenic mosquito strains, it is possible to visually distinguish heterozygote and homozygotes by the intensity of fluorescence, although human visual judgments may not be 100% accurate. Fluorescence quantification can be automated with much greater accuracy. Instruments such as the Complex Object Parametric Analyzer and Sorter (COPAS) XL have been used for high-throughput sorting of transgenic *Anopheles* and *Drosophila* larvae [8], based on expression of fluorescent transgenes (similar to FACS sorting of fluorescently-marked cells), and can distinguish heterozygotes from homozygotes based on empirically-optimized gating of transgene fluorescence levels in a rapid, high-throughput manner [26]. If care is taken to optimize construction of the four initial transgenic strains with regards to fitness, genetic background, insert location and fluorescence expression, rapid construction of an outcrossed MLA mosquito strain should be feasible because the desired genotypes can be automatically identified.

**Figure 8:**
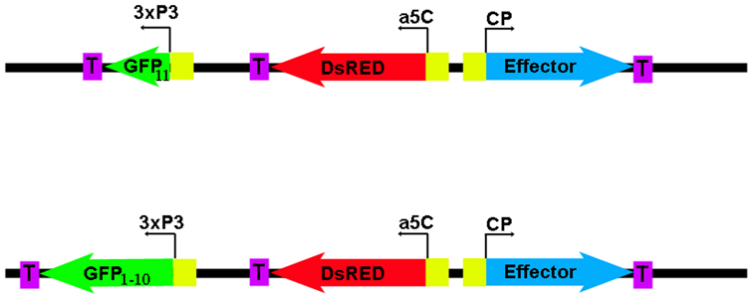
Potential strategy using a self-assembling fluorescent reporter protein to visually identify mosquitoes carrying 1 vs. 2 inserts at a specific genomic phi C31 location. Mosquitoes heterozygous for each individual transgene (identified by constitutive DsRED fluorescence) are crossed. F1 mosquitoes inheriting a transgene copy from each parent exhibit eye-specific (3xP3) GFP expression. When GFP-expressing F1 mosquitoes are crossed, all subsequent generations will be homozygous for the effector transgene. CP = bloodmeal-induced carboxypeptidase promoter, aC5 = constitutive actin5C promoter, T = terminator.

For some scenarios, it may be even easier to identify heterozygotes vs. homozygotes at a give locus. GFP can be split into 2 fragments (amino acids 1–214, or “GFP_1–10_”, and amino acids 214 to 230, or “GFP_11_”). By themselves, the individual fragments do not fluoresce, but when both fragments are simultaneously co-expressed, they self assemble into a functional fluorescent protein [27]. One could envision creating, at a specific genomic locus using the phi C31 system, two mosquito strains that carry transgene inserts that are identical for the effector gene and a constitutively-expressed marker gene (such as DsRED) to identify transgenic individuals. However, each strain will carry an additional transgene under a specific promoter (such as 3xP3); one strain carrying GFP_1–10_, and the other strain carrying GFP_11_. When crossed, only those individuals carrying both transgene construct variants will exhibit GFP fluorescence (Figure 8). When GFP-expressing individuals are crossed in future generations, all mosquitoes will carry a transgene insert at that locus, and all will be homozygous for the effector molecule. Only individuals possessing both variants will exhibit GFP expression, but at this point GFP is irrelevant. The split reporter system can also be used with other reporter molecules [27].

## Discussion

We think it is likely that the MLA strategy will be better suited to controlling Dengue virus rather than malaria. MLA strains will be much easier to generate and deploy for *Aedes* species compared to *Anopheles* species. *Aedes aegypti* effective population sizes are generally more manageable than *Anopheles*, although *Anopheles* populations do undergo significant reductions during the dry season [28]–[29] that could be exploited for manageable releases. In addition, *Aedes aegypti* adults tend to remain locally rather than migrating large distances [28], [30]. Equally importantly, *Aedes* are easier to bloodfeed and rear in large numbers, and their eggs can be desiccated and archived for long periods of time [31].

It is important to clearly identify the strengths and weaknesses of the MLA strategy compared to other active methods for transgene introduction currently under consideration. While important data may be obtained through the use of large contained field cages [32], active drive methods are impossible to test under true field conditions prior to the first actual release. If the transgene spreads, but does not reduce disease levels, or if unforeseen consequences result, it may be difficult or impossible to return to the unmodified situation. However, if the transgenic intervention is successful, the genetically-modified insects should spread spatially across the range of the vector, potentially leading to very cost-effective reduction or elimination of the disease across its range because additional effort or resources should not be required [14]–[15].

In contrast, MLA represents a potential method to test disease-modulating transgenes in the field for safety and efficacy, and offers a method to eliminate the transgenic organisms from the environment in the case of adverse effects, changing policies, or simply at the end of the trial. However, even if successful, MLA will not spread transgenes widely across a wide geographic area, but rather will lead to local increases in transgene frequency. This weakness will be exacerbated if the transgenes induce fitness costs. As such, outside of a test situation, MLA will be best suited to intervention attempts in defined geographic areas with restricted immigration from outside areas, similar to the constraints experienced by the sterile insect technique [23].

### Model limitations

Like all models, significant simplifying assumptions have been made that may not hold true in reality. We assume that the population is panmictic, not density-regulated, has discrete generations, does not exhibit spatial or age structure, and that environmental factors do not impact transgene characteristics. In addition, we do not address questions related to evolutionary impacts, epidemiological dynamics or feedback, or complex interactions between transgene fitness loads and parasite infection status.

## Methods

### Modeling of multi-locus traits

Theory has been developed to follow the spread of some types of multi-locus traits, but these efforts focused on the spread of conditional lethal or female-killing genes, and were based on lepidopteran genetics [21]–[22]. Since mosquitoes have only 3 pairs of chromosomes [31], it is unlikely that transgenic mosquitoes carrying many unlinked loci can be constructed. The model is a simple extension of the model outlined previously [12], expanded for 4 unlinked loci: α, β, χ and δ (total 8 inserts maximum per genome). As the following model simulations show, this number is adequate for reasonably rapid spread of transgenes into populations. If inserts induce cumulative fitness costs, increasing the number of loci beyond this level does not dramatically increase the efficacy of the strategy due to the multiplicative fitness cost experienced by released mosquitoes.

There are 2 possible alleles at each locus: α; Aa, β; Bb, χ; Cc, and δ; Dd, where uppercase denotes presence and lowercase denotes absence of the transgene at that locus. At each locus there are three possible locus-specific genotypes – thus, with four loci there are 3^4^, or 81 possible global genotypes (*G_k_*), where *k* = 1 to 81 and the number of transgene inserts per genome ranges from 0 to 8: aabbccdd, aAbbccdd, AAbbccdd, aaBbccdd……AABBCCDD

There are 16 possible gametes that can be formed: ABCD, ABCd, ABcD, ABcd, AbCD, AbCd, AbcD, Abcd, aBCD, aBCd, aBcD, aBcd, abCD, abCd, abcD, abcd.

The frequency of each genotype *G_k_* at generation t is *P_k_*_,_, which assuming random mating can be calculated from the allele distributions. The model assumes that fitness costs are the same for all inserts and that the costs of multiple inserts are assessed multiplicatively. Each genotype is assessed a fitness cost 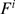, where *i* = 0 to 8 and *F* = the relative fitness of an individual carrying a single transgene insert. The fitness (*W*) of each genotype is calculated as 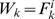. The proportional contribution of each genotype can be calculated as 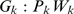. The average fitness of the population (*W_ave_*) is calculated as the sum of these terms 
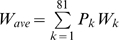
. Genotype frequencies at generation *t+1* are calculated as 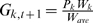.
